# Multimerization interactions between protein-inspired single-chain random heteropolymers

**DOI:** 10.1371/journal.pone.0349103

**Published:** 2026-05-28

**Authors:** Shayna L. Hilburg, Tianyi Jin, Priya Ganesh, Alfredo Alexander-Katz

**Affiliations:** 1 Department of Materials Science and Engineering, Massachusetts Institute of Technology, Cambridge, Massachusetts, United States of America; 2 Department of Chemical Engineering, Massachusetts Institute of Technology, Cambridge, Massachusetts, United States of America; National Institutes of Health, UNITED STATES OF AMERICA

## Abstract

Single-chain nanoparticles (SCNPs) are attractive for their ability to interact with or behave akin to native proteins. In this work, we use molecular dynamics simulations to examine how SCNPs which are assembled non-covalently due to the hydrophobic effect and monomers with negative Flory-Huggins interaction parameters (χ) in water can interact with other macromolecules. The occurrence of multimerization is characterized for a methacrylate-based random heteropolymer system with heterogeneous surfaces and sequence behaviors. The system shows two primary interaction modes: (1) adsorbing through side-chain interactions similar to protein oligomerization and (2) maintaining their single-chain structures or with only transient interactions. For cases in which adsorption does occur, the small, amphiphilic methyl methacrylate monomers are shown to enrich at the points of contact. Hydrophobic residues are typically present at the interface when adsorption is prolonged, while hydrophilic monomers associate in more transient inter-polymeric interactions. Finally, we demonstrate that polymer conformation of a single polymer sequence plays a large role in multimerization, while the variation among these conformations is statistically indistinguishable from the variation amongst different sequences.

## Introduction

Due to their enzymatic activity, biocompatibility, and unique mechanical properties, proteins have potential applications in a wide range of fields, from tissue engineering to sustainable packaging design [1,2]. Protein structure is strongly correlated with function, and depends on intramolecular interactions, including covalent linkages and hydrogen bonds [3,4]. Approximately 80 percent of proteins in the body operate in complexes, so the strength and specificity of inter-molecular interactions has high biological relevance [5]. Several diseases, including cancer, multiple sclerosis, and rheumatoid arthritis, have been linked to faulty protein-protein interaction networks [6]. While RHPs do not have well-defined secondary and tertiary structure like structured proteins, another class of proteins called intrinsically disordered proteins (IDPs), lacks a fixed three-dimensional structure. IDPs have much larger interactomes, or set of potential interactions, than proteins with fixed structures, since their dynamics and high adaptability increase the number of species with which they can interact [7]. With less chemical specificity, IDPs have a broader range of possible interactions. Changes in salt concentration, pH, temperature, and solvent can affect the strength of these interactions, thus limiting function of the protein. Statistically designed random heteropolymers (RHPs) have emerged as a potential solution to this problem, protecting proteins from losing activity in solvents which typically cause denaturation and chaperoning them to fold properly in cell-free synthesis conditions [8,9]. These RHPs consist of four methacrylate-based building blocks that vary in size, charge, and polarity to promote the formation of a heterogeneous surface that can interact well with proteins. RHPs have even been shown to exhibit protein-like behavior themselves; when embedded in a lipid bilayer, they can perform a similar function to proton channels in a cell membrane [10]. In recent work, this RHP was shown to function as an enzyme mimic by creating a hydrophobic environment around a charged residue [11].

Polymers interface with surfaces through a pattern-matching process in which segments most frequently associate with surfaces that contain complementary chemical interactions [12,13]. For SCNPs, the transition from intramolecular to intermolecular behavior therefore occurs upon interfacing with surfaces patterned in a compatible manner. Characterization of such molecular interactions could be leveraged in applications at synthetic or biological interfaces, including cellular, viral, and bacterial surfaces [14]. We previously studied the energetics and dynamics of RHP assembly through all-atom molecular dynamics (MD) simulations [15–21]. The heteropolymers were found to assemble into compact, single-chain nanoparticles (SCNPs) with heterogeneous surfaces with limited backbone mobility due to hydrophobic collapse in water. In non-aqueous environments, however, the RHPs remodeled, extending into solutions of intermediate polarity and in highly non-polar solvent, forming compact globules with opposite driving forces from aqueous SCNPs. Those RHPs have also been found to stabilize membrane proteins *ex membrane* by spanning the lateral surface of β-barrel proteins and reducing loop fluctuations [22]. These findings, in concert with the experimental observation of RHP behavior with proteins and lipid bilayers, create interest in further characterizing the rough energy landscape affording a complex set of intermolecular behaviors.

Herein, we assess such intermolecular behavior for our aqueous RHP systems. Specifically, we investigate their potential for multimerization when two identical RHPs are co-located in solution. The potential for oligomerization of these RHPs has been previously indicated by small-angle X-ray scattering [15]. The propensity for dimerization is shown to vary even for the same polymer sequence, with heterogeneous polymer surfaces and relatively glassy solution behavior of each RHP creating a set of energetic interactions within the system even more complex than those explored intramolecularly.

## Materials and methods

**Fig 1:**
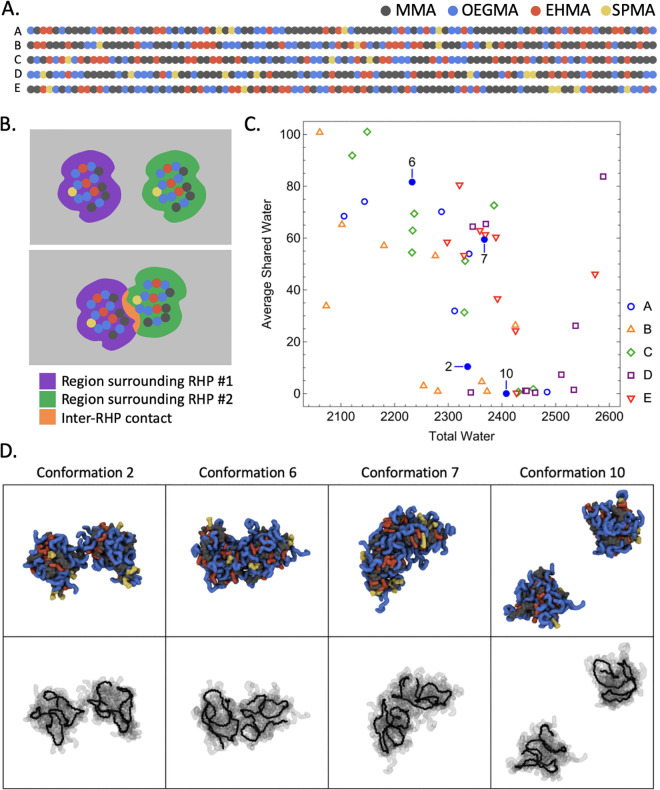
Determining intermolecular contact. **(a)** Five RHP sequences that are included in this work. **(b)** A schematic illustration of the inter-RHP contacts as the overlap between the regions surrounding two individual RHPs. **(c)** Varied amount of intermolecular contact shown through solvation data as Total Water versus Average Shared Water for each conformation. Points for Sequence A conformations 2, 6, 7, and 10 are plotted with filled blue markers, and snapshots from simulations of those corresponding conformations are shown in **(d)**. Atoms rendered in Gray belong to MMA monomers, Blue are OEGMA, Red are EHMA, and Yellow are SPMA. In the lower row of renderings, side-chains are translucent and the polymer backbone is shown, highlighting the lack of backbone integration. In all renderings, water and counterions are omitted for clarity.

The RHPs simulated in this study consist of methyl methacrylate (MMA), oligo(ethylene glycol) methacrylate (OEGMA), 2-ethylhexyl methacrylate (EHMA), and 3-sulfopropyl methacrylate (SPMA) monomers. A set of 5 sequences, as shown in Fig 1A, of the four-component methacrylate-based RHP—a subset of those simulated in Ref. [15] each 100 monomers in length with target MMA:OEGMA:EHMA:SPMA ratios of 50:25:20:5—were incorporated into dimerization studies in water. This particular RHP formulation has been experimentally shown to be highly versatile, including but not limited to stabilizing proteins [8,9], transporting protons across lipid membranes [10], and catalyzing terpene cyclization [11]. For each sequence, 10 separate systems were examined containing two identical RHP conformations previously equilibrated alone in water. Packmol [23] was used to place equilibrated RHPs from Ref. [15] at (20 Å, 20 Å, 20 Å) and (−20 Å, −20 Å, −20 Å) such that their center coincides with the center of a 160 Å × 160 Å × 160 Å cube containing 5000 SPC/E water molecules. The general Amber force field (GAFF) was used [24], and all monomers were assigned partial charges from restrained electrostatic potential (RESP) charges [25] using Gaussian 03 Revision D.01 [26]. The classical force field and fixed-charge model were intended to investigate the dominant physical interactions and general trends governing RHP association, rather than to provide a fully exhaustive or quantitatively exact description of all molecular details. Potassium counterions were added to balance the negative charge of the SPMA monomers and achieve an electrically neutral system. Amber 2019 [27] was used to perform a short minimization and a 2 ns equilibration at 1.0 bar. Each system was then run for 120 ns at 300 K and 1.0 bar. Sequence A conformations 7 and 10 were replicated for interfacial composition analysis an additional 4 times. In this study, we focused on the kinetics of oligomerization rather than equilibrium structures, and the 120 ns simulation window was used to characterize the dynamics.

AmberTools and various Python libraries were used for data analysis [27–30]. In order to calculate the number of shared water molecules between RHPs, *watershell* function of pytraj (from AmberTools) was used to calculate the number of water molecules contained within 5.0 Å (the second solvation shells) of both RHPs, represented schematically in Fig 1B. The percent of time adsorbed is the amount of simulation time in which at least 40 water molecules were shared between the two RHPs divided by the total amount of simulation time. This cutoff is chosen to reflect the two clusters shown in Fig 1C: cluster 1, characterized by high average shared water and low total water, corresponding to high contact formation (Conformation 6 and 7); and cluster 2, characterized by low average shared water and high total water, corresponding to low contact formation (Conformation 2 and 10). In order to determine the percent of residues that interact, the number of residues that contain an atom that comes within 10 Å of any atom of the RHP was determined, then divided by the total number of residues. Pytraj’s root-mean-square fluctuations (RMSF) function was used to calculate the mean RMSF of each residue’s backbone atoms over the last 40 ns of each trajectory. A contact between two residues is defined as a time point at which a heavy (non-hydrogen) atom from the first residue is within 10 Å of a heavy atom from the second residue. The interface composition was determined by the residue identities of atoms that appear within 10 Å of the other RHP. This quantity is weighted by time (i.e., atoms appearing for two time frames will count twice, atoms appearing for 5000 time frames will count 5000 times).

A Kruskal-Wallis H test [31] is applied to assess the sequence sensitivity of oligomerization. This nonparametric statistical test evaluates whether multiple samples originate from the same distribution. The dataset includes 10 independent conformations from each of five sequences, and the data are not normally distributed.

## Results and discussions

### Single-chain conformation impacts RHP-RHP interactions

Over the course of 120 ns of unbiased MD simulation, the intermolecular behavior between two identical RHPs in water has been characterized. On average, a relatively small percentage of the RHP atoms interact inter-molecularly, though the amount of time for which they adsorb to one another varies considerably (Table 1).

**Table 1:** Adsorption behavior between random heteropolymers. The mean and standard deviation of the percent of simulation time spent adsorbed and the percent of polymer atoms which interact, averaged over the 10 conformations of each sequence.

	Sequence A	Sequence B	Sequence C	Sequence D	Sequence E
Pct. of time adsorbed (%)	60.3 ± 38.6	47.4 ± 40.0	69.4 ± 37.5	34.2 ± 40.7	74.3 ± 32.1
Pct. of residues with any atom interactions (%)	15.3 ± 1.0	12.0 ± 1.1	14.1 ± 0.8	3.1 ± 0.5	10.7 ± 0.8
Pct. of residues with backbone interactions (%)	7.1 ± 7.3	4.0 ± 5.6	5.6 ± 5.3	1.5 ± 3.6	2.1 ± 2.3

**Fig 2:**
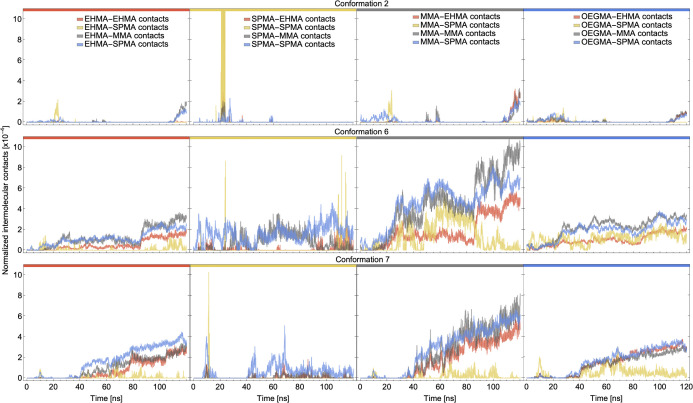
Intermolecular contact formation over time. Intermolecular contacts between two random heteropolymers over time for each monomer type. The number of contacts between residue types *A* and *B* has been normalized by a factor of a*b, where *a* is the number of atoms of residue type *A* and *b* is the number of atoms of residue type *B* in a single RHP. Each column of plots shows the contacts for a given residue in one of the RHPs with the monomers of the other RHP, as identified by line color where Gray are MMA, Blue are OEGMA, Red are EHMA, and Yellow are SPMA atoms in the second RHP. Plots are for sequence A conformations 2, 6, and 7, corresponding to transiently touching and strongly adsorbed intermolecular interaction behavior as seen in Fig 1.

Two distinct types of adsorption behavior are observed in Fig 1C-D, with either low total water content and high shared water content indicating RHP association (top left of graph in Fig 1C) or high total water and low shared water indicating high solvation of each chain independently (bottom right region of Fig 1B). Representative examples of these cases are rendered in Fig 1D from sequence A. The number of shared water molecules is a measure of interaction strength. The first category, exhibited by conformations 6 and 7, shows long-term contacts, whereby the polymer surfaces touch in a dumbbell-like morphology, in a fashion qualitatively similar to protein oligomerization. These interactions are dominated by surface association with minimum backbone remodeling. Inter-molecular contacts, separated and normalized by residue identity, are plotted for reperesentative conformations as a function of simulation time in Fig 2. In the second category, shown for conformations 2 and 10 with low shared water content and high total water content, there are nearly zero shared water molecules. Conformation 10 has so close to zero contacts in the 120 ns simulation that there are no features to be shown in a plot akin to those in Fig 2. For Conformation 2 (top of Fig 2), several transient associations occur during the simulation as the molecules diffuse through the system. Short-lived contacts occur through side-chain interactions, and few intermolecular contacts are seen between monomers of all types. In contrast, for the more highly-associated Conformations 6 and 7, data shown in the middle or bottom rows of Fig 2, respectively, EHMA, MMA, and OEGMA contacts increase steadily as the polymer reconfigures to assemble an internal structure dominated by hydrophobic interactions. Although there are only few SPMA monomers, they do show transient interactions with various other residues in those systems. Contact between multiple SPMA monomers is observed in some cases for each of the representative plots (second column of Fig 2), and can be attributed to counterion salt bridging through visual inspection. OEGMA, and to a lesser extent MMA, contacts also occur transiently in this system. However, EHMA contacts appear longer-lasting, correlating to more enduring adsorption. Stepwise contacts are observed, similar to adsorption onto a hard interface [19], suggesting the presence of a latent period during which the flexible side groups gradually approach and adsorb onto another RHP. Complete merging into a more spherical object is hindered by internal friction and the high energetic cost of backbone rearrangement [16], demonstrating converged radii of gyration upon interacting with other biomacromolecules [22]. Instead, side-chain interactions and rearrangements dominate. This internal friction is attributed to the glassy nature of PMMA backbone [32,33] and the high miscibility between PEG moiety and PMMA [20,34].

Single-molecule RHP assemblies have been shown to be relatively glassy in water, with atomic fluctuations of the side-chains far exceeding those of the polymer backbone [15,18,32], resembling the dynamics of the molten globule phase of native proteins. Through the assessment of backbone motion, using RMSF during the final 40 ns of simulation, we examine how adsorption can alter such vitrification. In Table 2, the backbone fluctuations in our systems are separated to independently compare the fluctuations of the backbone atoms for residues which have intermolecular interactions and those which do not. While even non-interacting residues show greater backbone fluctuations in our two-RHP systems compared to the single RHP characterized in Ref. [15], a much more appreciable difference is seen for the interacting residues. This finding tracks with some remodelling seen in strongly adsorbed dimerization behavior observed through visual inspection, indicating that intermolecular RHP interactions correspond to increased, albeit still limited, backbone rearrangement at the interface. Although, as previously discussed, the molecules are not unfolding or fully integrating, the RMSF data is evidence of some remodeling at the interface to optimize interfacial contact in adsorbed configurations.

**Table 2:** The root-mean-square fluctuations (RMSF) of random heteropolymer (RHP) backbones averaged over 10 conformations for polymers individually in solution (data from simulations in Ref. [15]) and as pairs in solution. Data for systems with two polymers is broken up into the residues which do and no not interact between polymer pairs.

	Mean RMSF of Backbone Residues				
	Sequence A	Sequence B	Sequence C	Sequence D	Sequence E
Single RHP	0.89	0.85	0.84	0.90	0.90
Multiple RHPs: Interacting residues	1.02	1.05	0.97	1.36	1.35
Multiple RHPs: Non-interacting residues	0.91	1.00	0.90	0.95	1.00

### Monomer chemistry impacts interactions

**Fig 3:**
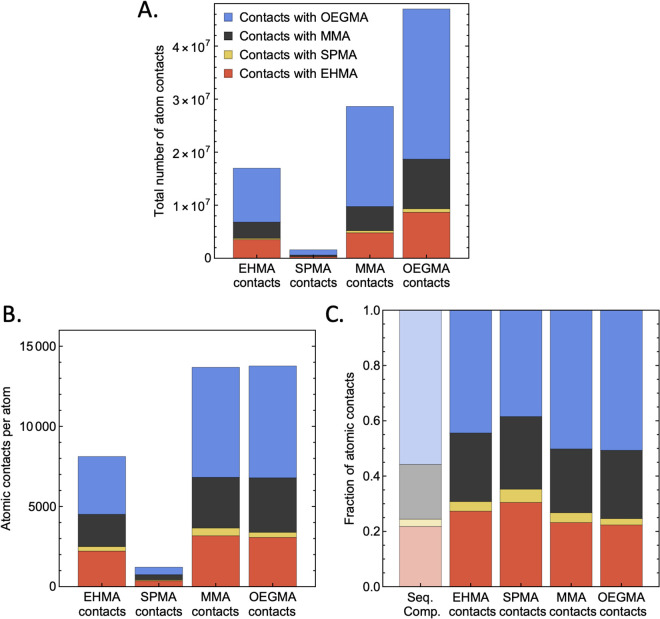
Average Sequence A intermolecular contact properties. Intermolecular contacts between two random heteropolymers by monomer averaged for 10 conformations of Sequence A. **(a)** Total number of contacts per residue type, **(b)** contacts per atom of each residue type, equivalent to the total number of contacts normalized by the number of atoms of a given monomer type within the polymer sequence, and **(c)** Contacts per monomer relative to overall sequence composition by atom, which is the same data but scaled from 0 to 1. Similar figures for the other 4 sequences are shown in S1-4 Fig in S1 File.

Initial analysis using the intermolecular contacts of selected conformations in Fig 2 prompted further investigation by monomer identity. Fig 3A shows the total number of atomic contacts for each residue type averaged over the 10 conformations of Sequence A and concludes that, by number, contacts with OEGMA residues dominate the interfacial interactions. As OEGMA has a significantly longer side-chain than the other monomer species, this finding is unsurprising. It is also clear that SPMA monomers barely interact with the other RHP, again unsurprising as SPMA is the least represented monomer in the sequences. However, more consequentially, upon normalization by the number of residue atoms to adjust for population quantity differences (Fig 3B), it can be seen that SPMA is in fact minimally present at the interface, even when accounting for its low frequency in the chain, due to its anionic charge making it the most hydrophilic. In comparison to the overall sequence composition (Fig 3C), OEGMA and EHMA representation in intermolecular contacts scale similarly to the mean behavior. MMA exhibits considerable enrichment as compared to its contribution to the chain by mass, especially considering its smaller side-chain size, making it less sterically accessible when compared to a long side-chain like OEGMA’s which can readily explore the space surrounding an RHP without backbone reaarrangement. Note that these trends hold for all 5 sequences (S1-S4 Figs in S1 File).

**Fig 4:**
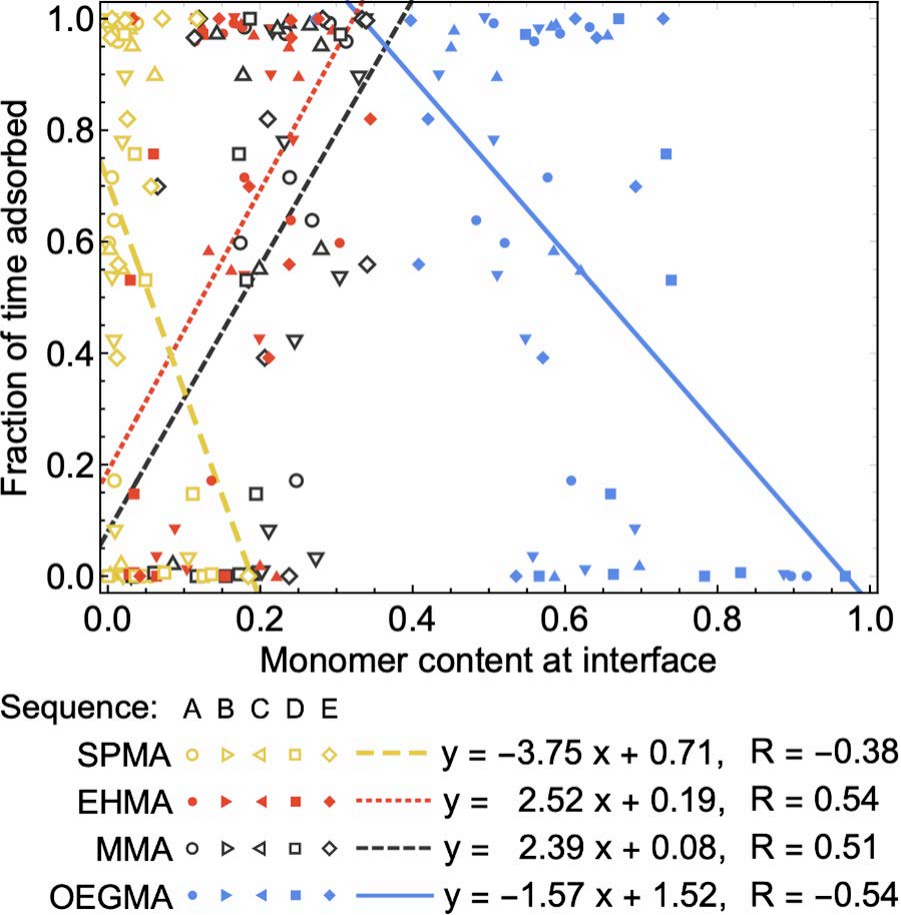
Relating time adsorbed to interfacial composition. Plot depicts the relationship between the type of chemical contact and length of adsorption for each monomer type. The monomer content at the interface between RHPs is plotted versus the fraction of simulation time spent adsorbed with line of best linear fit for each monomer type. Each datapoint contains information for a single conformation of one of 5 simulated sequences. The equation of linear regression for each monomer type and corresponding correlation coefficient are shown. Note that for each of the 50 conformations, there are 4 datapoints (1 for each monomer species).

To illuminate whether there is a correlation between interfacial composition and the longevity of adsorption, as was qualitatively observed in Fig 2, the fraction of time adsorbed was plotted versus the interfacial content of each monomer species for all 50 simulated configurations (Fig 4). While fraction of time varies considerably at intermediate content percentages for MMA, OEGMA, and EHMA, adsorption is more effected by extreme enrichment or depletion of the residues at the interface. For the hydrophobic monomers MMA and EHMA, particularly low interfacial presence precludes prolonged intermolecular adsorption, while high interfacial presence is correlated to more enduring contact. OEGMA interfacial contact shows the opposite trend, hypothesized to be due to the significant entropic penalty of restricting the long side-chain’s motion. SPMA interfacial content is extremely low in most conformations, regardless of time adsorbed.

### Sequence cannot determine oligomerization

**Fig 5:**
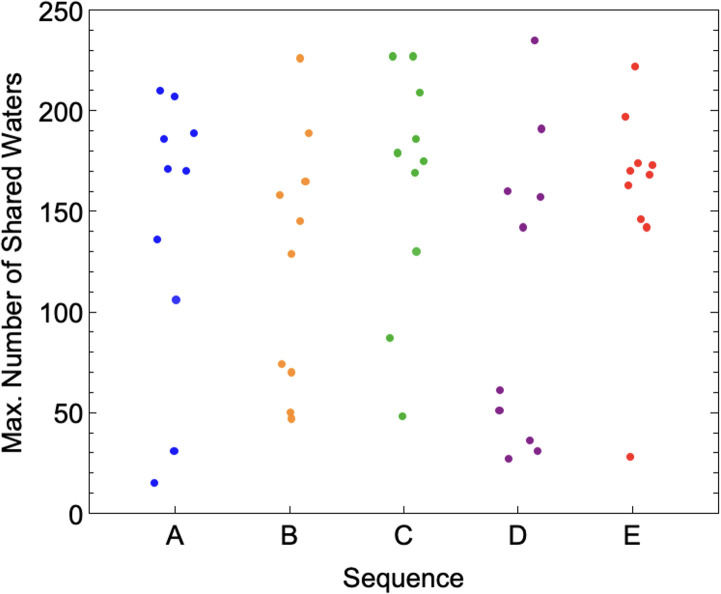
Variance within and between sequences. Maximum number of shared waters by sequence, as used for Kruskal-Wallis H test, p-value = 0.42. Each datapoint represents a single conformation of each sequence.

Each RHP conformation, as explored in prior work [15,16], has a distinct backbone topology despite having the same monomeric sequences, and therefore also presents unique surfaces towards the solvent. These different surface arrangements and energies, therefore, impact how the conformations interface. Highly varied fraction of time adsorbed and interfacial composition can be observed for different conformations of the same sequences in Fig 4. Variability of interfacial behavior between different starting structures of identical sequences is, in fact, great enough that it cannot be distinguished from the variability between entirely different sequences (interfacial composition shown for all 50 conformations in S5 Fig in S1 File). A Kruskal-Wallis H test was performed on datasets of the maximum number of shared water from each simulation (Fig 5). While median values varied between sequences, a p-value of 0.42 indicates that the analysis did not find statistically significant differences of the variances within and between sequences for these properties. The same analysis has been conducted for the fraction of time adsorbed to one another and the sum total of shared waters over the simulation for each simulation, with results depicted in S6 Fig in S1 File. The observed insensitivity of interfacial oligomerization to sequence reflects an observation previously reported for the hydration of single-chain RHP monomers, which was also found to be largely sequence-insensitive [35].

**Fig 6:**
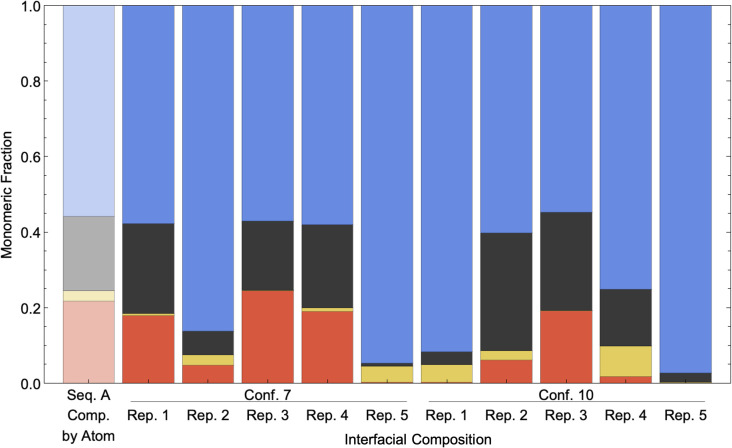
Interface composition for replicate simulations. Interface composition normalized to 1 across the five replicates of Conformations 7 and 10 of polymer sequence A. Each set of values for a given replicate/conformation is presented next to the overall composition of the respective chain. At the interfaces, MMA is shown in Gray, OEGMA is shown in Blue, EHMA shown in Red, and SPMA shown in Yellow. Note that normalization is to 1 regardless of amount of time interacting, and as such several conformation interfacial compositions are for very short periods of time including Rep. 2 and 5 in Conformation 7 and Rep. 1, 4, and 5 in Conformation 10.

This stochasticity can further be extended to simulation replicates. For Conformation 7 and 10 of Sequence A, a total of five replicates are conducted, starting from the same initial structures but seeded with distinct velocities. Fig 6 shows that variations in the interfacial composition across replicates are notable. It should be considered that interfacial composition was normalized to 1 regardless of amount of time in contact, meaning several of the compositions have limited data in their composition information due to short contact times including Rep. 2 and 5 in Conformation 7 and Rep. 1, 4, and 5 in Conformation 10. Indeed, those with short contact times have large OEGMA interfacial fractions, consistent with the observation above that OEGMA intermolecular contacts have been described above to occur in high proportion during short interactions, as shown in Fig 4. We would like to highlight that Rep. 2–5 for both conformations in Fig 6 are absent from Fig 4; however, the trends match well, demonstrating the robustness of this behavior. For those with longer contact times, the interfacial compositions are more varied and show stochasticity across different replicates.

## Conclusion

Throughout this work, we have demonstrated the stochastic behavior of random heteropolymer SCNPs in multimerization simulations. Despite containing chemically identical components, some systems interacted only passively for short periods of time while others showed inter-polymeric complexation. These diverse behaviors spanned between and amongst sequences, showing disparate adsorption mechanisms for different conformations of the same sequence. When adsorption is observed, it occurred as primarily surface and side-chain dominated interactions. Such strong adsorption between the RHPs increased polymer dynamics, particularly of the backbone for monomers involved in interactions at the interface. The behavior observed in these cases is in contrast to the slow dynamics previously observed for such molecules alone in aqueous solution [15,32], and offers insight into how complex interactions with heterogeneous protein surfaces could lead to adaptive RHP behavior which enables protein protection and mimicry [8,9,22,36,37]. From a compositional perspective, SPMA infrequently existed at the interface. While OEGMA had the most interfacial contacts, when normalized by composition, MMA was notably enriched at the interaction sites. Hydrophobic interfacial content from such MMA or EHMA residues was also shown to correlate with multimer longevity. The hydrophobic interfaces are reminiscent of protein oligomerization and are consistent with prior speculation of how the RHPs may interact through hydrophobic patches. Further work must explore how disparate molecules–rather than two identical polymers with the same sequence–interact to find the limitations of such trends in such heterogeneous systems. However, given the current dataset, we can envision that the trends of multimerization are likely tunable based on surface composition and thus, monomer composition, which can be leveraged in future applications and optimization targeting particular behavior [38].

## Supporting information

S1 File S1–S6 Fig. (PDF)
