# Supplementary material for: Multimerization interactions between protein-inspired single-chain random heteropolymers
Source: PLoS One. 2026 May 28;21(5):e0349103. doi: 10.1371/journal.pone.0349103 (PMC13218474; doi:10.1371/journal.pone.0349103)

Supporting Information: Multimerization Interactions Between Protein-Inspired Single-Chain Random Heteropolymers

Shayna L. Hilburg, Tianyi Jin*, Priya Ganesh, Alfredo Alexander-Katz
Email Address: herryjin@mit.edu

S1-S4 Fig. Intermolecular contacts between two random heteropolymers by monomer for sequences B, C, D, and E akin to main text Figure 3 (for sequence A). Data is totaled for 10 conformations of that single polymer sequence. (a) Total number of contacts per residue type, (b) Number of contacts per residue atom, equivalent to the total number of contacts normalized by the number of atoms of a given monomer type within the polymer sequence, and (c) Contacts per monomer relative to overall sequence composition are shown for each.
Monomer identity is shown as MMA in gray, OEGMA in blue, EHMA in red, and SPMA in yellow.

S1 Fig, Sequence B:

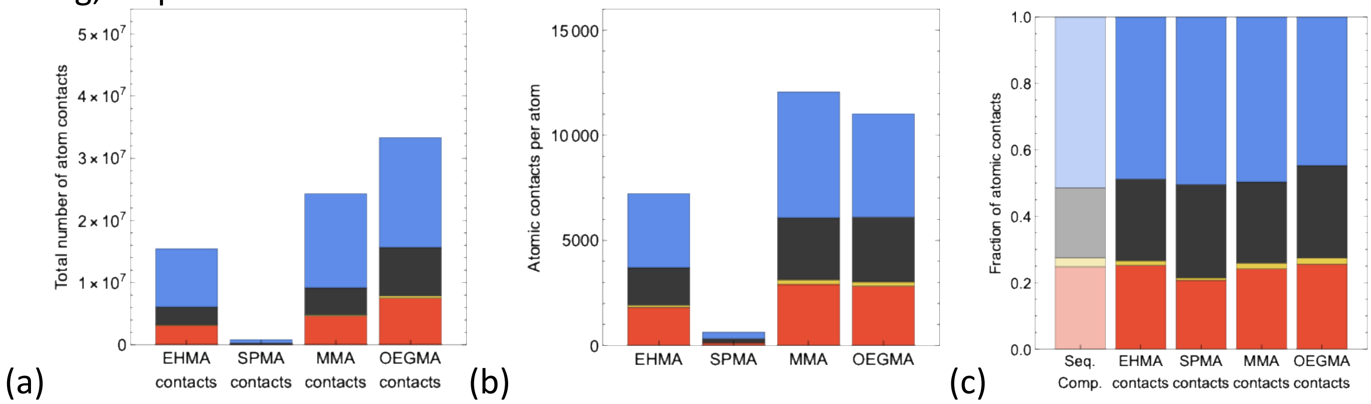

S2 Fig, Sequence C:

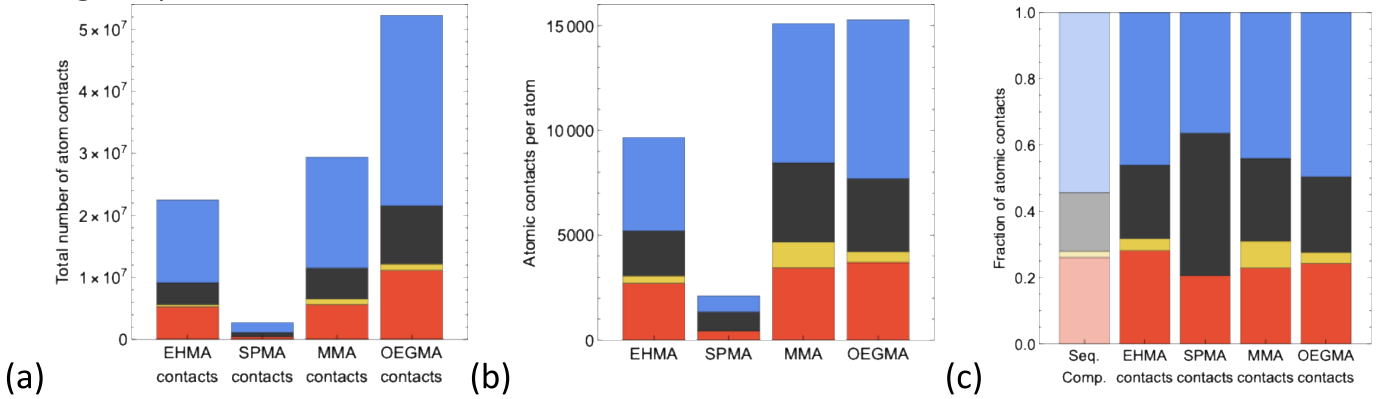

S3 Fig, Sequence D:

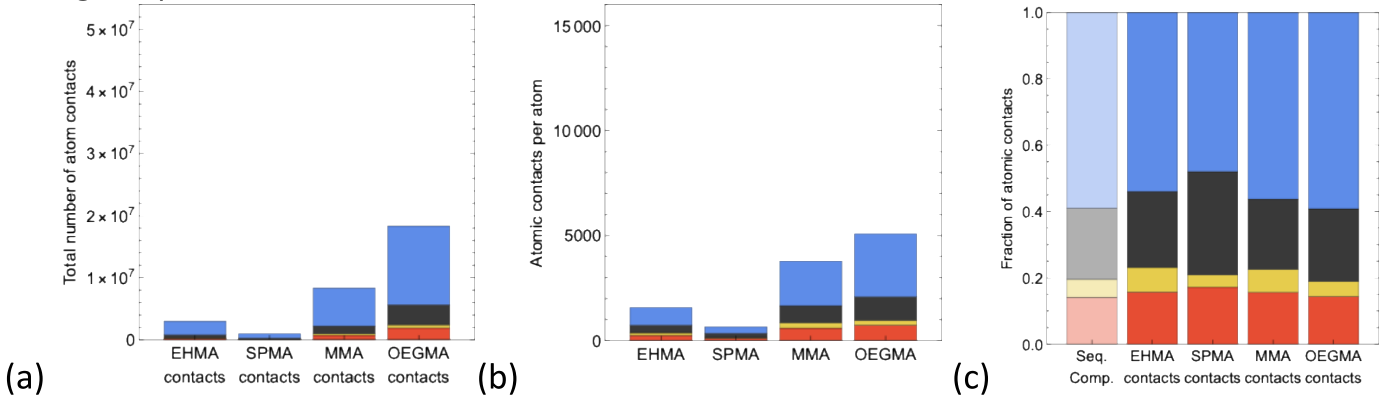

S4 Fig, Sequence E:

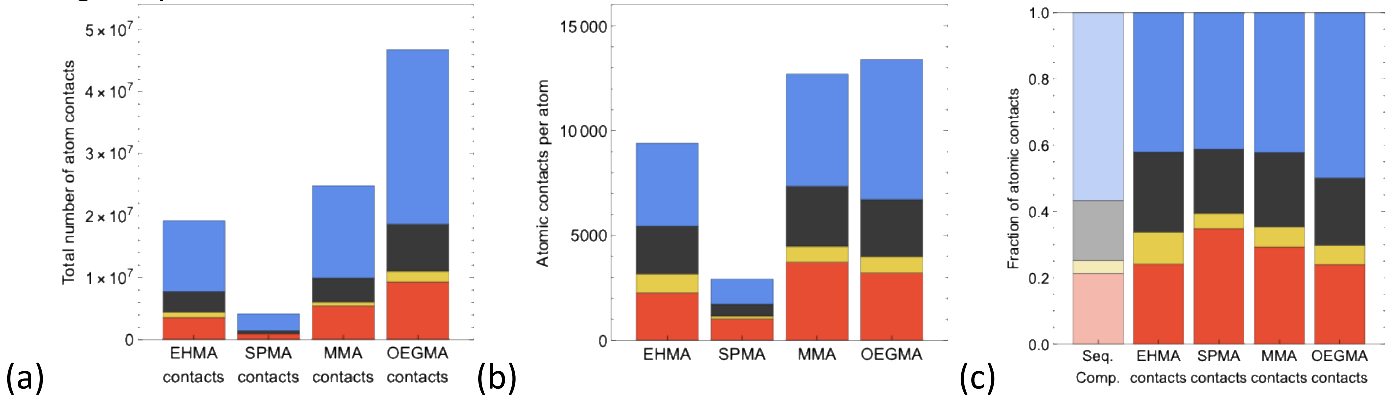

S5 Fig. Interface composition normalized to 1 for the individual conformations for each polymer sequence. Each set of values for a given sequence is presented next to the overall composition of the respective chain by number of atoms of each monomer type. It is separated for each of the 10 conformations simulated. Note that normalization is to 1 regardless of amount of time interacting, and as such several conformation interfacial compositions are for very short periods of time. Monomer identity is shown as MMA in gray, OEGMA in blue, EHMA in red, and SPMA in yellow. Akin to main text Figure 6 which showed replicates of Sequence A conformations 7 and 10.

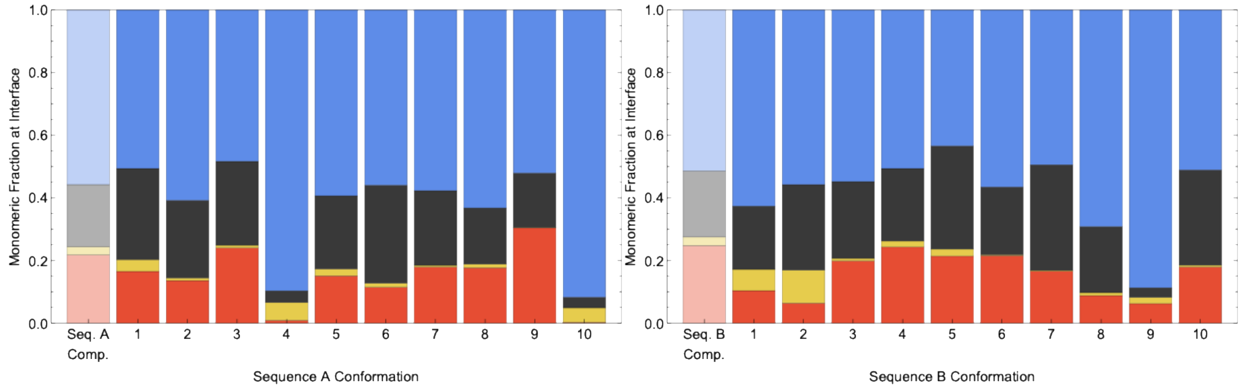

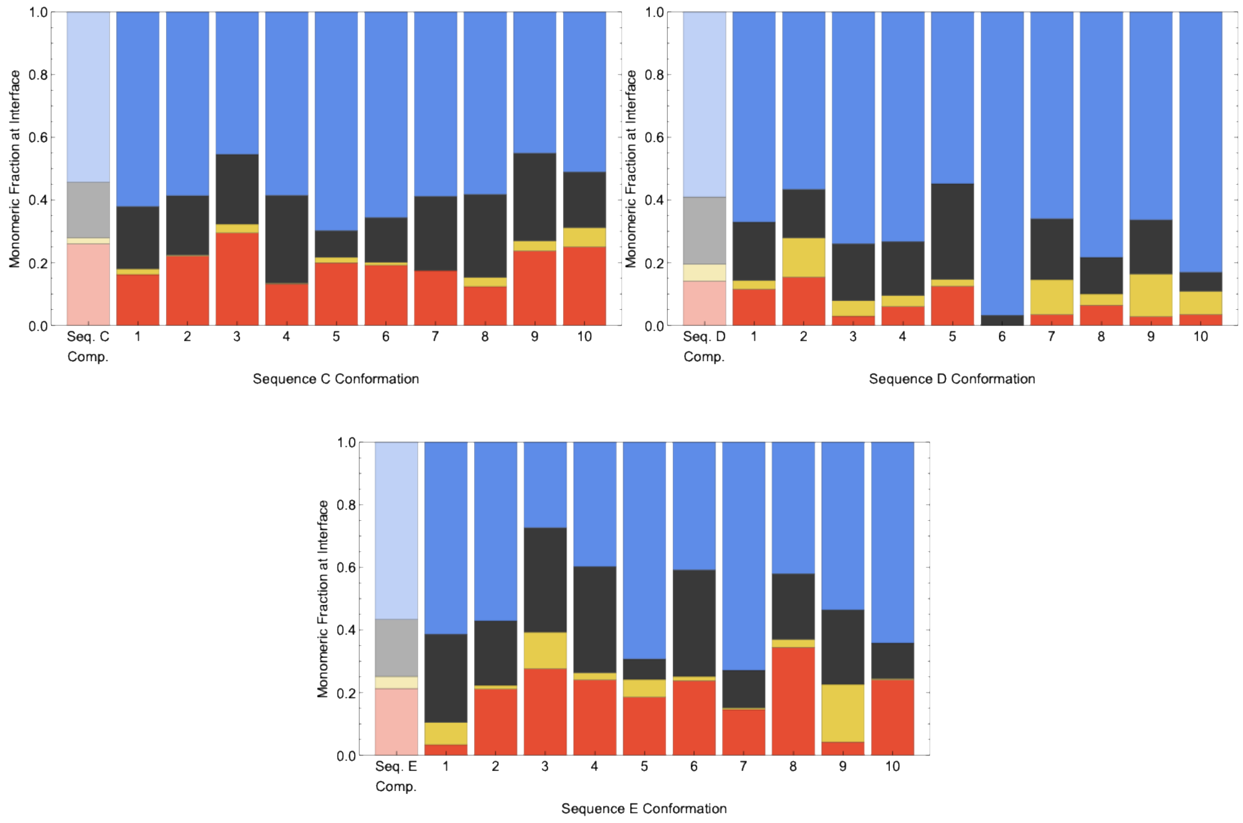

S6 Fig. Kruskal–Wallis H test datasets describing: (top) the sum total of shared waters over the simulation for each conformation, and (bottom) the fraction of time stuck. While mean values varied between sequences, p values of 0.30, and 0.20, respectively, indicate that the analysis did not find statistically significant differences of the variances among and between sequences for these properties

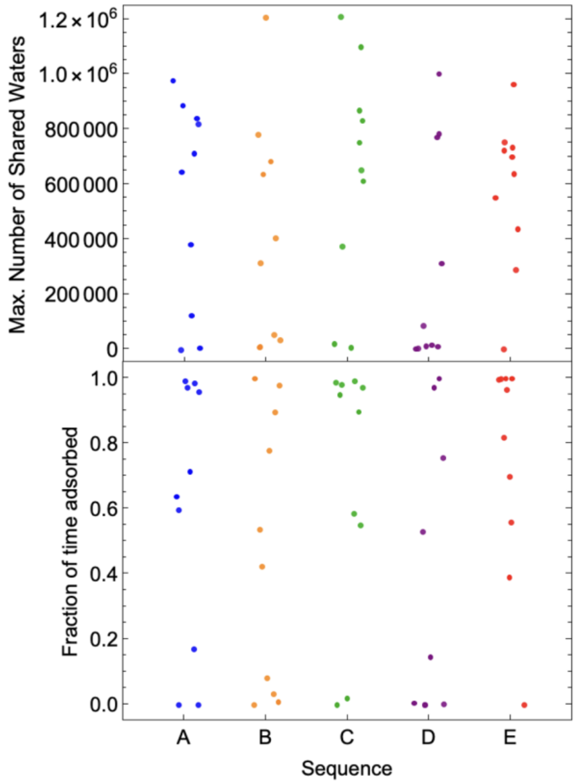

Supplement: S1 File — (PDF) [file pone.0349103.s001.pdf]
